# Comparing acoustic and satellite telemetry: an analysis quantifying the space use of *Chelonia mydas* in Bimini, Bahamas

**DOI:** 10.1098/rsos.231152

**Published:** 2024-01-10

**Authors:** Emily E. Hardin, Joshua A. Cullen, Mariana M. P. B. Fuentes

**Affiliations:** Marine Turtle Research, Ecology and Conservation Group, Department of Earth, Ocean & Atmospheric Science, Florida State University, Tallahassee, FL 32304, USA

**Keywords:** acoustic tracking, biotelemetry, satellite tracking, sea turtle, spatiotemporal distribution, utilization distribution

## Abstract

Passive acoustic and Argos satellite telemetry are common methods for tracking marine species and are often used similarly to quantify space use. However, data-driven comparisons of these methods and their associated ecological inferences are limited. To address this, we compared temporal durations, spatial resolutions, financial costs and estimates of occurrence and range distributions for each tracking approach using nine juvenile green turtles (*Chelonia mydas*) in Bimini, Bahamas. Tracking durations were similar, although acoustic tracking provided higher spatiotemporal resolution than satellite tracking. Occurrence distributions (95%) estimated from satellite telemetry were 12 times larger than those from acoustic telemetry, while satellite range distributions (95%) were 89 times larger. While individuals generally remained within the extent of the acoustic receiver array, gaps in coverage were identified. These gaps, combined with the lower accuracy of satellite telemetry, were likely drivers for the larger satellite distributions. Costs differed between telemetry methods, with acoustic telemetry being less expensive at larger sample sizes with a previously established array. Our results suggest that acoustic and satellite telemetry may not provide similar inferences of individual space use. As such, we provide recommendations to identify telemetry methods appropriate for specific study objectives and provide discussion on the biases of each.

## 1. Background

Knowledge of how species use space through time can help address key ecological questions by providing information on animal movement patterns [1], geographic range [2] and migration pathways [3], as well as connections to ecologically important habitats [4,5]. Additionally, data on space use in association with environmental drivers can provide insights into habitat and resource selection [6]. Combined, these data can be used to forecast space use in relation to specific changes, such as climate change impacts, and can thereby aid adaptive management practices [7,8]. This is particularly important within coastal nurseries and foraging grounds, as these areas provide essential benefits to individuals (e.g. availability of food, safety from predators) and therefore require targeted and effective management based on robust ecological and spatial data [9,10]. Efforts to develop and improve methods to estimate an animal's space use have led to a myriad of innovative research techniques [11–14]. With several approaches available, identifying the most appropriate method for a specific research question is necessary, particularly as technology continues to advance.

Visual transect surveys [15], aerial surveys [16], mark-recapture studies [17], stable isotope analyses [18] and biotelemetry tracking studies [19,20] are a few of the survey approaches frequently used to determine species' space use. Due to the logistical constraints associated with observing marine species *in situ,* as well as recent technological developments, the use of biotelemetry devices has grown rapidly in recent years, with acoustic and satellite telemetry emerging as common approaches across many aquatic taxa [14,21]. Tracking data from these methods have successfully addressed high-priority ecological questions and informed conservation and management efforts [22–24]. However, while each of these approaches has unique benefits, they also have specific limitations, biases, and assumptions that need to be considered when designing a study.

Passive acoustic telemetry monitors tagged individuals via an array of underwater receivers [25]. Internally or externally applied transmitters (depending on the target species) emit coded acoustic signals that are recorded as the tagged individual passes within the detection range of a receiver. This method is therefore constrained and biased by the design (i.e. density and extent) of the receiver array, as well as receiver detection ranges and tag transmission power [25–28]. The exact locations of individuals are typically unknown, although they can be identified within 1 m depending on the receiver set-up [25]. Regardless, this method has been used successfully to track species that remain in localized areas during one or more life stages, such as teleosts, crustaceans, elasmobranchs and chelonids [21,29]. Receivers continuously monitor for nearby acoustic transmissions, enabling acoustic telemetry to provide fine-scale data with high spatial (1–100 s of metres) and temporal (less than 1 min) resolutions [25,30]. Given that acoustic signals only transmit well through water, this method is advantageous for species that spend considerable time underwater, but is less suited for seabirds or species that exhibit basking or hauling-out behaviours [28].

Alternatively, traditional satellite telemetry (i.e. via platform transmitter terminals) uses external transmitters that relay data to overhead satellites as the tagged individual surfaces, which is then used to derive geographic locations of that individual [28]. It is therefore well-suited for species that are at the ocean-air interface frequently, but less feasible for marine fishes or species that make long, deep dives [31]. Location accuracy of satellite telemetry is highly dependent on the type of transmitter. Fastloc-GPS transmitters quickly acquire and transmit GPS ephemeris data from which highly accurate positions can be derived, but their higher costs can be a deterrent, with researchers often opting to increase sample sizes by deploying more of the less-expensive Argos-only transmitters [32,33]. Accuracy of these more widely-used Argos transmitters is affected by the number of overhead satellites present and the amount of time the transmitter has to communicate with the satellites [34,35]. This often results in location errors greater than 1.5 km [30]. However, the main advantage of satellite telemetry is that it is not spatially constrained to an array (like acoustic telemetry) and can therefore track individuals undertaking large-scale movements or migrations [3,36], although it can also be used to track individuals on a smaller scale (e.g. within foraging areas) [37].

Acoustic telemetry is often considered to be a low-cost tracking approach, particularly when compared to satellite telemetry [25]. However, discussions of associated costs are generally limited to the transmitters themselves, while other costs associated with telemetry, particularly the cost of data retrieval, are often overlooked or difficult to quantify. Acoustic telemetry requires large initial costs to install an array, after which regular maintenance and physical data retrieval are required [25,38,39]. While the costs associated with this can be quite large, the costs of the transmitters themselves are in the order of hundreds of US dollars [38,39]. Argos and Fastloc-GPS satellite transmitters are expensive, costing thousands of dollars, with additional fees to access the Argos network to retrieve the associated data [39,40]. Fastloc-GPS transmitters can also be used as data loggers, which store data on-board the device, but the tag must be retrieved from the individual, which has additional costs [35]. Detailed assessments of the full financial costs associated with each telemetry method are needed to compare pertinent trade-offs.

Despite their differences, acoustic and satellite telemetry are both frequently used to quantify and interpret the space use of individuals and populations within the marine environment [41–43]. However, data-driven comparisons that assess whether these two common biotelemetry methods can be used to make similar ecological inferences are lacking. To our knowledge, only three studies have yet attempted to quantitatively compare space use metrics derived from acoustic and satellite telemetry [39,44,45]. All three studies assessed Fastloc-GPS satellite telemetry and all reached varying conclusions regarding the applicability of each approach. As such, it is still unclear whether acoustic and satellite telemetry provide similar estimates of individual occurrence and range distributions. Furthermore, there have been no studies to date that have assessed acoustic telemetry with the more ubiquitous Argos satellite telemetry, warranting a need for comparisons between these two commonly used methods. Findings from these comparisons can be used to inform decisions on the most appropriate method to use when accounting for experimental design and research objectives, and can help managers properly interpret findings from telemetry studies with considerations for biases and limitations [44].

To address this gap in knowledge and systematically compare space use estimates between telemetry methods, we simultaneously tracked nine juvenile green turtles (*Chelonia mydas*) with both passive acoustic and Argos satellite telemetry within a foraging area in Bimini, Bahamas. Multiple aspects of both tracking methods, including the temporal durations and resolution, spatial extent and resolution and financial costs associated with each tracking method were compared. Our objective was to assess whether similar inferences can be made from both tracking methods when taking into consideration all of the inherent limitations, biases and assumptions that each encompasses. To do so, we compared individual occurrence and range distributions to (1) evaluate how each telemetry method affects the interpretation of space use of foraging turtles in Bimini, and (2) assess the applicability of each telemetry method for projects aiming to quantify current and future space use of marine species.

## 2. Methods

### 2.1. Study site

**Figure 1.**
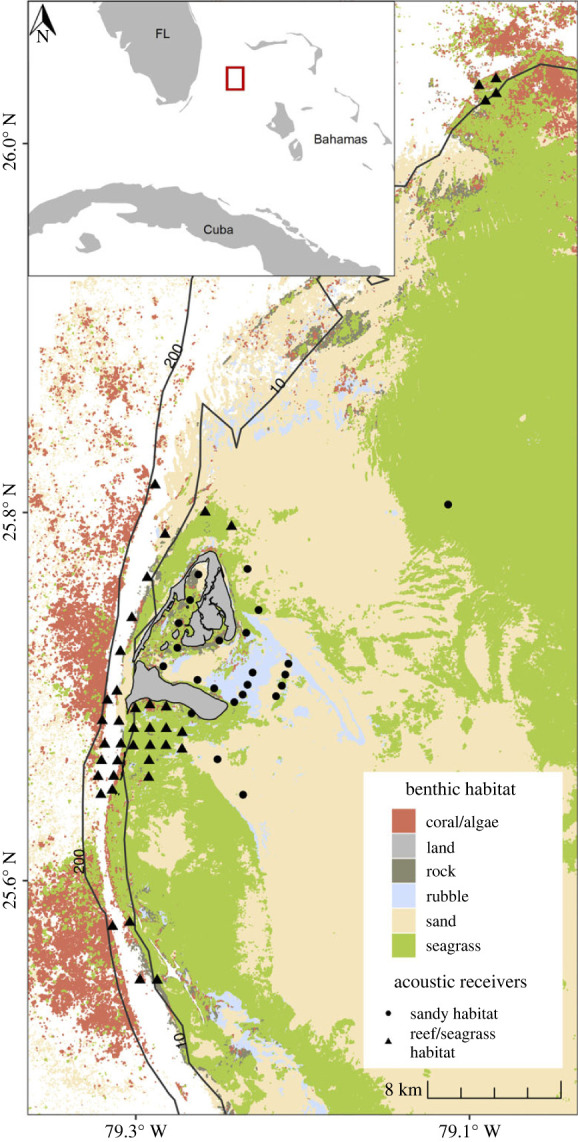
Study area map of Bimini, Bahamas with benthic habitat (white = unclassified) and locations of passive acoustic receivers (*n* = 63) shown. Based on range tests, receivers in reef or dense seagrass habitat were assigned detection ranges of 185 m, while receivers in sandy habitat or the shallow lagoon were assigned detection ranges of 350 m. Benthic habitat data were obtained from the Allen Coral Atlas 2020, and bathometric isobars of 10 m and 200 m are shown.

This study took place in Bimini, Bahamas (figure 1), located approximately 86 km off the coast of Miami, Florida, USA (FL) in the western Great Bahamas Bank (25°44′ N, 79°16′ W). Bimini consists of two main islands (North and South Bimini) situated in a triangular shape with a semi-enclosed lagoon [46]. Benthic habitat surrounding the islands includes fringing coral reefs, mangroves, shallow seagrass and unconsolidated sand habitat [46–48]. Bimini's waters are an important nursery for an abundance of species, specifically juvenile sharks [49,50], and are a prominent foraging area for juvenile green turtles [51,52].

### 2.2. Turtle capture

Vessel-based haphazard, unmarked, non-linear transect (HUNT) surveys [53] were conducted in May 2017 to locate juvenile green turtles. Turtles were hand-captured via the ‘rodeo method’ [51,54] and brought on board for standard work-up. Morphometric measurements were taken including standard and curved carapace length and width, plastron length, head width and tail length (all ± 0.1 cm), as per Gillis *et al*. [42]. Body weight (± 0.1 kg) was also taken with a hanging balance (PESOLA AG, PHS100). If not present, passive integrated transponders (PIT tags; Biomark GPT12) were inserted sub-dermally in one front flipper and Inconel flipper tags (National Band and Tag Company, Style 681) were applied to the trailing edge of both front flippers for individual identification. Turtles were equipped with both acoustic and satellite transmitters (see subsequent sections) and were released within 200 m of their initial capture site.

### 2.3. Acoustic tracking

Turtles were equipped with V13 acoustic transmitters (69 kHz, 50–130 s delay interval, 513 d battery life, Innovasea [previously Vemco], Bedford, Nova Scotia, Canada). Transmitters were affixed to the dorsal posterior marginal scutes with electrician tie-wraps fitted through 3 mm diameter drilled holes and secured with epoxy putty (Sonic-Weld®) [55]. Acoustic detections were monitored throughout Bimini and nearby islands by an array of 63 VR2W acoustic receivers (figure 1) maintained by the Bimini Biological Field Station Foundation. Receivers were originally placed to capture movements of sharks and rays around the islands, within the shallow lagoon, and particularly within seagrass beds to the south of Bimini (figure 1). Individuals in this study were monitored from May 2017 until no more detections were logged. Receiver detection ranges were defined as the distance at which 50% of expected acoustic signals were detected [27,56] and were calculated by a binomial logistic regression from data provided by range tests. Briefly, range tests were conducted at two representative locations between August 2015 and June 2019, one in deeper water (12 m) on the edge of a coral reef, and one in shallower water (2–3 m) in an open sand habitat [47,50]. Four test transmitters placed 1 m above the seafloor at increasing distances (0, 250, 500, 750 m) from a receiver at each test site were used to estimate receiver detection ranges, found to be approximately 185 m in reef habitat and 350 m in sandy habitat.

Acoustic detection data were filtered to remove possible false detections, including double detections, detections prior to transmitter deployment, and a single detection that occurred with no other detections within one hour prior to or after it [47,50,57]. Detections within the first 24 h following an individual's release were discarded to allow for acclimation after capture [55,58]. Additionally, detections made between 04 May and 06 May 2017 (57 h total) were removed due to a disruption in coverage of one-third of the receivers while being maintained. Abacus plots were created for each individual turtle with the VTrack package [59] in R [60] to visually inspect detections over time and to identify when transmitters were possibly dislodged or shed [61,62].

To account for the unknown true location of an individual within the detection range of a receiver [25,28], we reassigned detection locations to fall randomly within the receiver's detection range as weighted by the detection probability kernel per habitat [41,63]. Receivers in reef or dense seagrass habitat were assigned a 185 m detection range, while those in sandy habitat or within the shallow lagoon were assigned a 350 m range, based on range tests. This provided a more realistic representation of the natural variance that would occur in turtle locations, as opposed to all locations occurring at the coordinates of the receiver. Any relocated detections placed over land were reassigned again until no points over land remained. Reassigned locations were used to calculate centres of activity (COAs) at 30 min time steps following the mean-algorithm position method [64] with the VTrack package in R [59,65].

### 2.4. Satellite tracking

SPOT6-287 (Argos) satellite platform transmitter terminals (PTTs; approx. 543 d battery life, Wildlife Computers, Redmond, Washington, USA) were also attached to each turtle concurrently with the acoustic transmitters following protocols by Seney *et al*. [66] using Power-Fast/Sonic-Weld epoxy putty [66]. These transmitters use the Argos satellite network to relay data to satellites from which geographic locations are derived via the Doppler shift [67]. Data were collected through December 2017 and downloaded from the Wildlife Computers data portal. The Argos system provides location accuracy for each observation as location classes (LCs), which have associated error estimates of <250 m for LC 3, 250–500 m for LC 2, 500–1500 m for LC 1, and greater than 1500 m for LC 0 [68]. Argos does not provide error estimates for LC A and B but experimental measures of accuracy have shown LC A to be variable and LC B to have the greatest error of the aforementioned classes [30,31,34,69]. LC Z are considered invalid locations [68] and were removed for this study's analysis, as well as locations recorded within the first 24 h of tag deployment to allow for an acclimation period. Duplicate transmissions, as well as implausible locations at the beginning of individual tracks, were removed.

### 2.5. Space use estimation

To comprehensively assess the tracking method's influence on the interpretation of individual space use and movement, we estimated both occurrence distributions and range distributions, collectively referred to here as ‘utilization distributions' (UDs). The occurrence distribution (OD) quantifies the uncertainty of an individual's movement path and can essentially provide a measure of how well each telemetry method estimates the movement of the individual [70,71]. The range distribution (RD) quantifies the predicted future space use and is synonymous with the traditional definition of an individual's home range [70–72]. Both ODs and RDs provide important yet distinct information on individual space use [71]. Data from each tracking approach were analysed via commonly used methods and in a manner consistent with the structure of the data. As such, the measures of occurrence and range distributions between telemetry methods do not provide all-else-equal comparisons to each other or to any ‘accurate’ measure of space use. Rather, our study uses the analytical approaches from other real-world studies to provide a comparison of how these commonly used methods may produce differing or similar interpretations of space use while incorporating their inherent assumptions and limitations, such as array design, resolution of data, and more.

#### 2.5.1. Occurrence distributions

We estimated individual 95% and 50% occurrence distributions (ODs) using dynamic Brownian Bridge Movement Models (dBBMMs) with the move package in R [73,74]. The dBBMM, an expansion of the Brownian Bridge Movement Model [75,76], works well for data sets of low temporal frequency [77], and provides a measure of the certainty of the movement pathway within the observed study period [70]. Additionally, it allows for a dynamic, rather than constant, Brownian variance of motion $(\sigma_{m}^{2})\;$ along the track in user-defined intervals, which allows the model to capture changes in behaviour throughout the track [78]. This interval, known as the sliding window, was set to the number of locations equivalent to approximately 24 h in order to capture subtle changes in behaviour as well as diel behaviours [78].

COA positions were used in the dBBMMs for the acoustic telemetry ODs. For the satellite data, a continuous-time correlated random walk was fit within a state-space model (SSM) using the aniMotum package in R [79] to account for location error of the raw data at the observed time interval. This model handles irregular sampling frequencies well and uses semi-major and -minor axis lengths, as well as ellipse orientation of Kalman filtered errors, to estimate ‘true’ locations quickly and reliably [80]. A conservative speed filter of 2 m s^−1^ was applied, and filters for angles and distances of outlier locations were set to 15°–25° and 1500–3000 m, respectively [37,80,81]. Fitted locations that overlapped with land were removed prior to any further analysis. Final fitted locations from the SSM were then used for satellite telemetry dBBMM ODs.

Since the acoustic COAs were calculated at 30-min time steps, the dBBMM window for the acoustic ODs was set to 49 observations (equivalent to approximately 24 h), with margins of 15 observations. ODs were not calculated for individuals with fewer than 49 COAs. For the satellite telemetry data, the median time step between transmissions was approximately 2 h, so the dBBMM window was set to 13 observations, with margins of 3 observations. If tracks contained gaps in data longer than 24 h, any variances associated with that section of the track were excluded from the dBBMM calculation [73]. Location errors for the acoustic telemetry data (COAs) were estimated to be the mean distance from the receiver to the reassigned detection locations for all detections used to calculate a particular COA. For satellite telemetry data, the location errors were taken to be the standard errors (SE) provided by the SSM output from the aniMotum R package, which propagated location uncertainty over both stages of the analysis. The package provides the SE in both the north-south and east-west directions, of which the smaller of the two errors was selected as the input for the dBBMM. Any location with a SE over 3 km was discarded.

#### 2.5.2. Range distributions

We also calculated 95% and 50% range distributions (RDs) with optimally weighted autocorrelated kernel density estimators (AKDE) using the ctmm R package [82] to estimate predicted space use per individual turtle [83]. Acoustic COA positions and raw satellite positions were first assessed for outliers based on a speed filter of 2 m s^−1^. The ctmm package was then used to fit several continuous time movement models (CTMM) to the data of each individual to estimate autocorrelation and positions, where the model with the lowest Akaike information criterion (AIC) score was selected per individual [82]. To produce results that are reflective of and consistent with previous published literature, particularly acoustic studies [62,84], location errors were not incorporated into the CTMM and resulting RD estimates. Weighted AKDEs were estimated to reduce bias from irregular sampling frequencies and locations over land were not removed, but land boundaries were incorporated into the AKDE function.

### 2.6. Space use comparisons

The dBBMMs and AKDEs were used to first estimate individual acoustic and satellite telemetry occurrence and range distributions (UDs; 95% and 50%), respectively, using all available data from each tracking method (referred to hereafter as ‘full temporal duration UDs'). Any portions of the UDs that fell over land were removed. The areas of the 95% and 50% UDs were calculated and compared between tracking methods with a Bayesian t-test with the BEST package in R [85], which provides a probability that the difference in space use estimates by telemetry method is greater than 0. The Bayesian model assumed a t-distribution to account for outliers and used an uninformative, broad prior [85]. Boxplots were used to visualize differences in mean UD size (utilizing R package ggbreak [86]). To assess the degree of agreement between each individual's acoustic-derived and satellite-derived UDs, we calculated the Bhattacharyya's Affinity (BA) overlap index for both 95% and 50% UDs, with the resulting value ranging from 0 (no overlap) to 1 (identical UDs) [87]. UDs were then estimated for only the dates when both acoustic and satellite transmitters were active to allow for comparisons across the same temporal period (referred to hereafter as ‘matching temporal duration UDs'). For the satellite UDs, any transmissions that occurred during the 57 h at the beginning of May when acoustic receivers experienced disruption were removed to accurately match the temporal duration of the acoustic UDs. Additionally, to assess how longer tracking durations may affect UD estimates, we compared the areas and overlap indices of matching and full temporal duration UDs for each individual with a Bayesian estimation, again using uninformative, broad priors [85]. Tracking durations of acoustic and satellite telemetry were compared with a Bayesian estimation as well.

Since passive acoustic telemetry is spatially limited by the receiver array, while satellite telemetry is not, we assessed the degree to which the individuals in this study used (or may use) space beyond the detection range of the passive acoustic array. For this, we calculated the overlap of the full temporal duration satellite 95% and 50% ODs and RDs and the detection range of the array as the proportion of the UD falling outside the array range. This percentage was calculated based on both the 50% (standard detection range definition) and 1% (maximum detection range) detection probability range of the receivers. Based on range testing, this equated to approximately 185 m and 520 m, respectively, for reef and seagrass receivers and 350 m and 1100 m, respectively, for sandy habitat receivers (see §2.3). Additionally, to further investigate whether individuals were using space beyond the boundaries of the array, as opposed to potential gaps in receiver coverage within the extent of the array, we calculated the minimum convex polygon (MCP) of the receivers and then calculated the proportion of the full temporal duration satellite UDs falling outside the receiver MCP [88]. To further compare acoustic and satellite telemetry, an exploratory analysis was conducted to compare ODs between methods when only satellite locations from within the 1% acoustic array detection range were used (see electronic supplementary material, analysis).

### 2.7. Cost analysis

The approximate costs associated with projects using acoustic and satellite telemetry were compared across several scenarios, which considered two different tracking durations (6 months and 12 months), four sample sizes (1, 5, 10 and 20 tracked individuals), and acoustic (V13) and satellite (Argos) transmitters. While not used in our study, we also included the costs associated with Argos-linked Fastloc-GPS, as we recognize that the use of these transmitters in tracking studies is growing [35,89]. Expenses related to capturing turtles for transmitter application, such as boat fuel, personnel time and travel/lodging at a field site, were not included in the cost analysis, since these expenses were similar regardless of whether satellite or acoustic transmitters were being applied. For all scenarios, we included the cost of the transmitter, as well as the costs of materials needed for transmitter application, such as epoxy or hardware. Battery life was similar between the satellite and acoustic transmitters used in this study (approx. 543 and 513 d, respectively), and as such were not taken into consideration, although this could impact final costs of projects depending on the sizes of tags required. For satellite telemetry scenarios, the tariff required for continued use of the Argos satellites was included. Acoustic telemetry scenarios included estimated costs associated with maintenance and data retrieval of a 40-receiver array, including personnel costs (see electronic supplementary material, methods, table S1). Maintenance costs included periodic receiver replacement costs (annual replacement of 10% of receivers) and annual receiver battery costs. Additionally, we estimated the costs associated with installing a 40-receiver passive array. Detailed information regarding how we define cost estimates is included in electronic supplementary material, methods.

## 3. Results

Nine juvenile green turtles (mean ± s.d.; SCL = 45.2 ± 7.1 cm; mass = 12.1 ± 6.8 kg) were tagged simultaneously with both acoustic and satellite transmitters in May 2017 (table 1). Seven of the individuals were tracked successfully with both technologies, while Turtle B was only tracked successfully with satellite telemetry and Turtle F with acoustic telemetry.

**Table 1.** Acoustic and satellite telemetry tracking information for juvenile green turtles tracked in Bimini in 2017. SCL (cm) is the standard straight carapace length. Tracking duration (d) is defined as 24 h post-release until the individual's last detection/transmission; acoustic tracking durations exclude 57 h span between 05/04 and 05/06 when 30% of receivers were inoperable. Processed locations refer to centre of activity (COA) locations for acoustic telemetry and post-state-space model (SSM) locations (listed first) and post-continuous time movement model (CTMM) locations (listed second) for satellite telemetry.

ID	SCL (cm)	mass (kg)	release date	tracking duration (d)		raw detections/ locations		processed locations/day	
				acoustic	satellite	acoustic	satellite	acoustic	satellite
A	56.2	24.8	5/2/17	80	74	3529	344	10	3/3
B	40.2	9.4	5/3/17	*NA*	234	*NA*	1651	*NA*	1/5
C	49.6	16.1	5/2/17	237	53	6365	90	6	1/1
D	38.9	5.6	5/3/17	102	100	3723	649	6	6/5
E	37.6	5.7	5/3/17	42	130	231	1000	1	3/3
F	41.9	6.5	5/2/17	81	*NA*	2854	*NA*	9	*NA*
G	38.7	7.5	5/2/17	68	152	3139	1178	14	5/6
H	51.0	14.5	5/3/17	8	20	46	129	2	5/5
I	52.4	18.8	5/3/17	81	186	385	1253	1	3/5
$\bar{x}$	**45.2**	**12.1**		**87**	**119**	**2534**	**787**	**6**	**3/4**
**SD**	**7.1**	**6.8**		**67**	**71**	**2193**	**573**	**5**	**2/2**

### 3.1. Acoustic and satellite tracking

**Figure 2.**
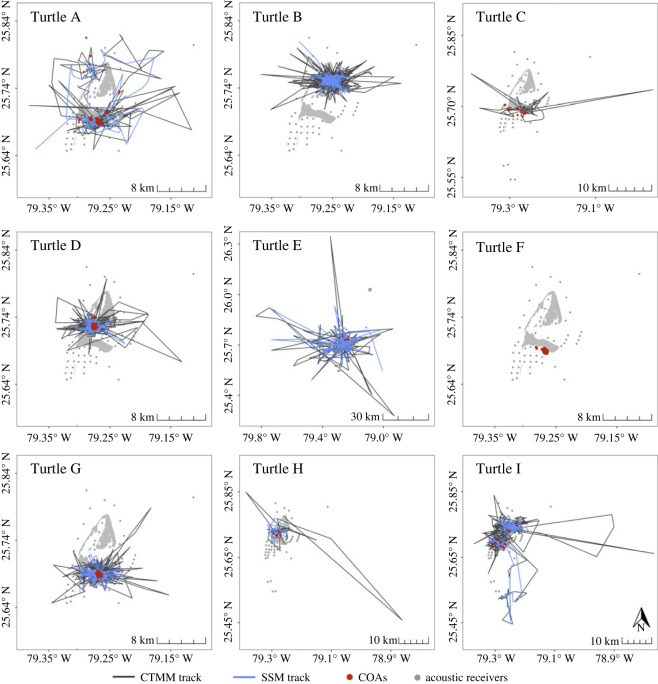
Post-continuous time movement model (CTMM) satellite tracks (dark grey lines), post-state-space model (SSM) satellite tracks (blue lines) and acoustic detection centres of activity (COAs; red circles) for nine juvenile green turtles tracked in Bimini, Bahamas in 2017.

Acoustic telemetry tracking durations (*n* = 8) were on average 87 d (range: 8–237 d), with 20 272 total detections logged across 19 unique receiver stations throughout the study area (table 1). Most of the detections (73%) were made at a single receiver station located in an area previously identified as having high densities of turtle grass and green algae [51]. After using the reassigned locations to estimate COAs (figure 2), individuals had a mean of 598 acoustic locations each, with an average of 6 locations per day (table 1). The mean location error radius of COAs was estimated to be 216 ± 118 m.

Individuals successfully tracked with satellite telemetry (*n* = 8) transmitted locations on average for 119 d (range: 20–234 d; table 1). There were 6294 raw satellite transmissions after initial filtering, with 6% of locations being assigned to LC 3 and 2 (Argos estimated location error: <250–500 m). After fitting the SSM to the satellite data to be used with the dBBMMs (figure 2), 45% of the estimated, corrected locations intersected with land and were subsequently removed. Additionally, 99 locations with SEs exceeding 3 km were removed from further analyses, resulting in an average of 373 satellite locations per individual (3 per day) with a mean location SE of 436 ± 452 m (table 1). After fitting individual CTMM models (see electronic supplementary material, tables and figures, table S2) to the satellite data to be used for AKDEs, the predicted tracks (figure 2) provided an average of 4 locations per day per individual. For each individual, there were differences between the tracking durations of the two methods; however, the mean difference between methods was 35 days (95% credible interval: −54.7–120).

### 3.2. Occurrence distributions

**Figure 3.**
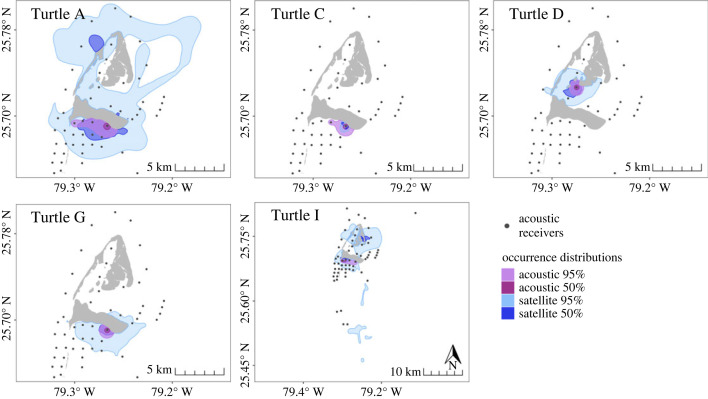
Full temporal duration 95% and 50% occurrence distributions (ODs) of individual turtles tracked with both acoustic and satellite telemetry in Bimini, Bahamas. ODs were derived from dynamic Brownian Bridge Movement Models using the full tracking extent (range: 53–237 d) for each method in Bimini, Bahamas.

**Figure 4.**
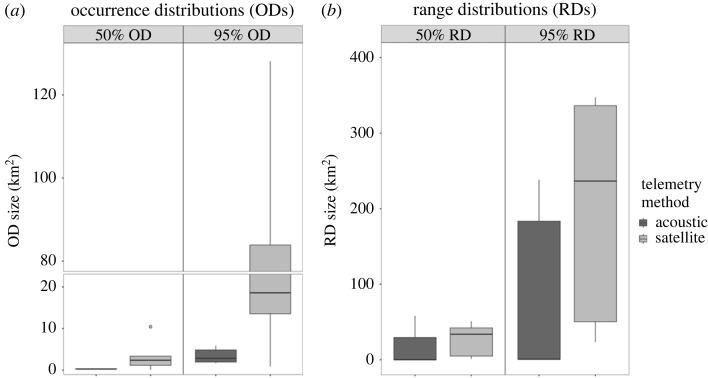
Comparisons of full temporal duration acoustic and satellite (*a*) occurrence distribution (OD) and (*b*) range distribution (RD) sizes for the five individuals with utilization distributions estimated from both methods. Dark horizontal bars indicate the median OD or RD size, with boxes showing the 25^th^ to 75^th^ percentile range. Vertical lines extend to the minimal and maximum values within 1.5 times the interquartile range, with outliers indicated by circles. Note the break in the y-axes and differing scales.

Turtles E and H had too few COA positions (*n* = 36 and *n* = 13, respectively) to calculate acoustic ODs via dBBMM and thus, similarly to Turtles B and F, were excluded from comparisons between acoustic and satellite ODs. For the remaining five turtles, full temporal duration acoustic ODs were smaller than satellite ODs for all individuals except Turtle C (figure 3). On average, satellite 95% ODs (mean ± SE; 49.02 ± 24.47 km^2^) were approximately 11.5 times larger (range: 0.3–22) and satellite 50% ODs (3.48 ± 1.83 km^2^) were 10 times larger (range: 0.3–25) than the respective acoustic 95% ODs (3.44 ± 0.84 km^2^) and 50% ODs (0.30 ± 0.03 km^2^) (figure 4*a*). There was an 89.3% probability that satellite 95% ODs were larger than acoustic 95% ODs, and an 88.4% probability that satellite 50% ODs were larger than acoustic ODs. The average BA overlap index comparing individual 95% acoustic and satellite ODs was 0.53, signifying a moderate level of agreement between ODs (table 2). The 50% ODs showed a lower degree of overlap, with an average BA index of only 0.16. When comparing ODs derived from only the period of time when both transmitters were active, the same trends were observed (see electronic supplementary material, tables and figures, figures S4, S5*a*, table S3).

**Table 2.** Overlap index values comparing full temporal duration acoustic and satellite utilization distributions. Bhattacharyya's Affinity index values range from 0 (no overlap) to 1 (identical).

ID	occurrence distributions		range distributions	
	50%	95%	50%	95%
A	0.13	0.47	0.72	0.64
C	0.10	0.64	0.01	0.01
D	0.18	0.54	0.02	0.03
G	0.28	0.69	0.14	0.07
I	0.14	0.33	0.47	0.71
$\bar{x}$	**0.16**	**0.53**	**0.27**	**0.29**
**SD**	**0.07**	**0.14**	**0.14**	**0.16**

There were no considerable differences in the sizes of the individuals' full and matching temporal duration ODs (see electronic supplementary material, tables and figures, figures S6, S7). Turtles A, C, and D were tracked for two to 184 days longer with acoustic telemetry than satellite telemetry and there was only a 52.5% and 52.7% probability that their acoustic 95% and 50% full temporal duration ODs, respectively, were larger than the matching temporal duration ODs (see electronic supplementary material, tables and figures, figure S6). Turtles E, G, H, and I were tracked for 12 to 104 days longer with satellite telemetry than with acoustic telemetry and similarly did not show substantial differences in OD estimates when including all tracking days (see electronic supplementary material, tables and figures, figure S7). Full temporal duration 95% and 50% ODs had only 47.8% and 36.2% probabilities, respectively, of being greater than the matching temporal duration ODs. The BA overlap indices comparing satellite and acoustic full temporal duration 95% and 50% ODs had a 61.8% and 64.4% probability, respectively, of being greater than the matching temporal duration overlap indices, indicating that increased tracking durations did not notably increase agreement of OD estimates between tracking methods.

**Figure 5.**
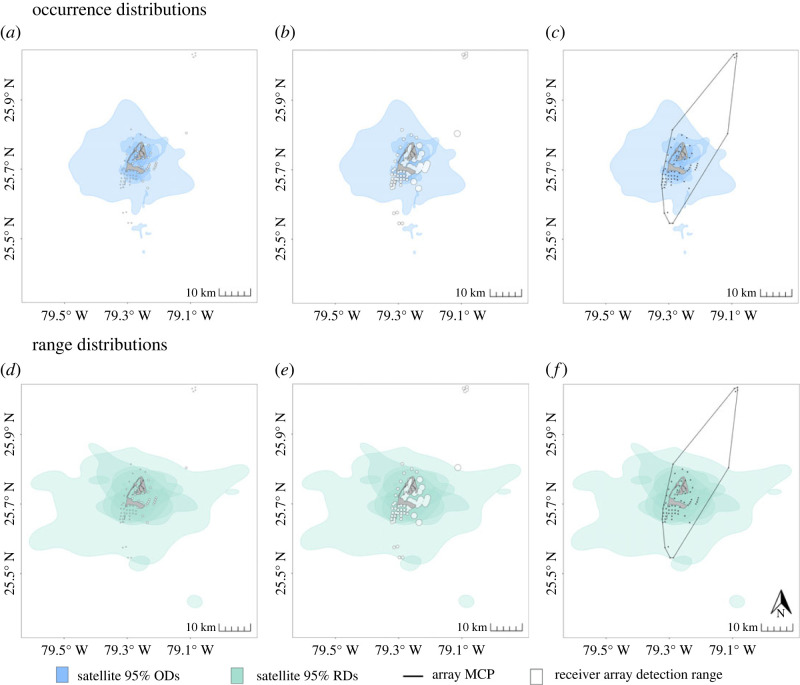
Spatial overlap between full temporal duration satellite 95% occurrence distributions ODs (*a*–*c*) and range distributions RDs (*e–f*) and the acoustic receiver array extent at (*a,d*) 50% detection probability and (*b, e*) 1% detection probability. Spatial overlap between the 95% ODs and RDs and the array MCP shown in (*c*) and (*f*), respectively.

All individuals in this study had occurrence distributions that extended beyond detection ranges of the acoustic receivers. At a 50% detection probability, the total coverage of the array was 13.09 km^2^ and, on average, 90% (range: 66–99%) of individual full temporal duration satellite 95% ODs fell outside the detection range of the receivers (figure 5*a*). Satellite 50% ODs were similar, with a mean of 85% (range: 56–100%) of the ODs occurring outside of the detection range (see electronic supplementary material, tables and figures, figure S8*a*). At the maximum possible detection range (1% detection probability), the extent of the array increased to 95.09 km^2^. Only one individual (Turtle C) had a satellite 95% OD that fell completely within the detection range of the receivers. On average, 46% (range: 0–91%) and 27% (range: 0 –57%) of satellite 95% and 50% ODs, respectively, still fell outside the detection ranges (figure 5*b*; see electronic supplementary material, tables and figures, figure S8*b*). With the exception of Turtle E, turtles were likely using space within gaps in receiver coverage as opposed to using areas beyond the extent of the array. On average, only 11% of the full temporal duration satellite 95% ODs extended beyond of the array's MCP, with Turtle E being the only individual to have a substantial amount of estimated movement (65% of satellite OD) outside the array bounds (figure 5*c*). Every individuals' 50% ODs fell within the array MCP (see electronic supplementary material, tables and figures, figure S8*c*). Restricting satellite data to only those locations that occurred within the 1% detection range of the receiver array did not significantly impact OD estimates and further supports the findings of the main study (see electronic supplementary material, analysis, figures S1, S2, S3).

### 3.3. Range distributions

**Figure 6.**
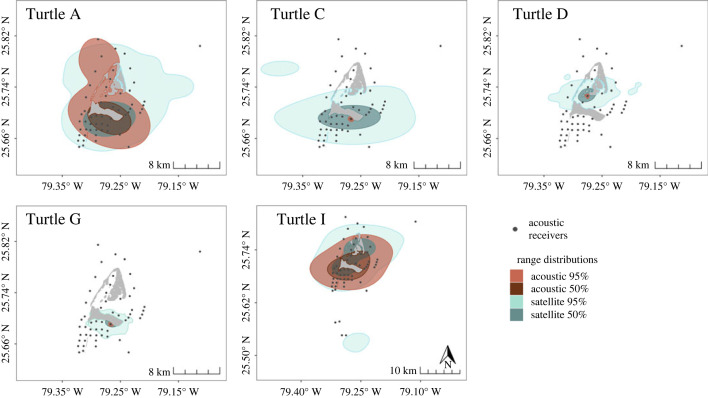
Full temporal duration 95% and 50% range distributions (RDs) of individual turtles tracked with both acoustic and satellite telemetry in Bimini, Bahamas. RDs were derived from autocorrelated kernel density estimators and estimated per individual using the full tracking extent (range: 53–237 d) for each method.

Similar to the occurrence distributions, Turtle E and H did not have enough acoustic data to calculate AKDEs, and therefore only Turtles A, C, D, G, and I were included in comparisons of range distributions (RDs). It should be noted that Turtle I had a low acoustic effective sample size and, as such, the acoustic home range of this individual had large confidence intervals and should be interpreted with care. The full temporal duration satellite RDs were larger than acoustic RDs (figure 6), with satellite 95% RDs (198.82 ± 68.99 km^2^) being on average 89 times larger (range: 1–305) than acoustic 95% RDs (84.70 ± 52.21 km^2^), with an 81.5% probability of being larger (figure 4*b*). Satellite 50% RDs (26.70 ± 10.02 km^2^) were on average 84 times larger (range: 0.9–315) than their acoustic counterparts (17.61 ± 11.66 km^2^), but with a 67.3% probability of being larger (figure 4*b*). BA overlap indices comparing full temporal duration acoustic and satellite RDs were low (table 2), with an average overlap of 0.29 for 95% RDs and 0.27 for 50% RDs. Similar trends were observed for matching temporal duration RDs (see electronic supplementary material, tables and figures, figures S5*b*, S9, table S3).

Of the turtles that were tracked longer with acoustic telemetry, Turtle A had a full temporal duration acoustic RD that was larger than its matching temporal duration acoustic RD; however, Turtles C and D showed no appreciable differences in RD estimates (see electronic supplementary material, tables and figures, figure S10). Overall, using the full acoustic tracking duration produced 95% RDs that had a 69.7% probability of being larger and 50% RDs that had a 68.9% probability of being larger than those estimated from the matching temporal duration data. Small differences were seen in satellite RD estimations for those individuals tracked longer with satellite telemetry, except Turtle H, whose full temporal duration RD was 13 times larger than its matching temporal duration RD (see electronic supplementary material, tables and figures, figure S11). Overall, full temporal duration satellite 95% RDs had only a 52.5% probability (and 50% RDs had a 47.8% probability) of being larger than satellite RDs derived from matching temporal duration data.

Similar to ODs, all individuals had full temporal duration satellite RDs that extended beyond the detection range of individual acoustic receivers. When considering a 50% detection probability of receivers, an average of 95% (range: 89–99%) of 95% RDs and 90% (range: 73–97%) of 50% RDs fell outside of the detection range of the receivers (figure 5*d*; see electronic supplementary material, tables and figures, figure S8*d*). At the maximum detection range of 1% probability, 68% (range: 38–95%) of 95% RDs and 37% (range: 2–55%) of 50% RDs fell outside the receiver detection range (figure 5*e*; see electronic supplementary material, tables and figures, figure S8*e*). While a large portion of predicted space use fell outside the individual receiver ranges, only 33% (range: 1–78%) and 5% (range: 0–25%) of 95% and 50% satellite RDs, respectively, fell outside of the MCP of the acoustic array (figure 5*f*; see electronic supplementary material, tables and figures, figure S8*f*).

### 3.4. Cost analysis

The estimated costs associated with each tracking technology varied depending on the scenario being considered (table 3). At small sample sizes (*n* = 1–5), the costs of individual satellite transmitters and Argos service were lower than the costs of acoustic array maintenance and data retrieval when considering an existing receiver array. Fees associated with accessing Argos satellite telemetry data were $378 per 6 months, compared to $10 800 over the same period for acoustic array maintenance and data retrieval. As such, tracking a single individual using acoustic telemetry was 5 and 2 times more expensive than tracking with Argos-only and Fastloc-GPS, respectively, in the short-term and 8 and 4 times more expensive, respectively, in the long-term. When tracking up to five individuals, costs became more comparable between Argos-only and acoustic tracking, with acoustic telemetry being 1.2 (at 6-months) and 1.8 (at 12-months) times more expensive than satellite tracking with Argos-only tags. Fastloc-GPS, however, was considerably more expensive than acoustic telemetry at samples sizes of five individuals or more.

**Table 3.** Total estimated costs associated with satellite and acoustic telemetry across varying temporal and sample size scenarios. Monetary amounts are USD. Values include all associated costs, including transmitters, materials, and labour costs. Acoustic telemetry values include maintenance and data retrieval costs.

duration	6 months				12 months			
sample size	1	5	10	20	1	5	10	20
acoustic with existing array	$11,325	$13,425	$16,050	$21,700	$21,725	$24,225	$26,850	$32,100
acoustic with array installation	$101,525	$103,625	$106,250	$111,900	$111,925	$114,425	$101,525	$122,300
satellite (Argos-only)	$2,303	$11,515	$23,030	$46,060	$2,681	$13,405	$26,810	$53,620
satellite (Fastloc-GPS)	$5,403	$27,015	$54,030	$108,060	$5,781	$28,905	$57,810	$115,620

As the number of tracked individuals increased, acoustic telemetry became the least expensive option when considering the use of an existing array. At a sample size of 10 individuals, Argos-only and Fastloc satellite telemetry were 1.4 and 3.4 times more expensive than acoustic telemetry, respectively, for a 6-month tracking period. However, for 12 months, the costs associated with Argos-only satellite telemetry and acoustic telemetry were equivalent. When increasing the sample size to 20 individuals, Argos-only and Fastloc satellite tracking were 2 and 5 times more expensive than acoustic telemetry, respectively, over a 6-month period. For a 12-month project, the differences were less, with Argos-only and Fastloc being 1.7 and 3.6 times more expensive than acoustic. Acoustic array maintenance and data retrieval costs remain relatively constant regardless of the number of individuals being tracked, so the lower costs of acoustic transmitters outweighed the more expensive satellite transmitters for projects with larger sample sizes (greater than 10). Installation of a 40-receiver acoustic array, including labour but not including array design and range testing, was estimated to be approximately $90 000 and added considerable expenses under all scenarios (table 3).

## 4. Discussion

The results from our study showed that passive acoustic and Argos satellite telemetry did not provide the same inferences of juvenile marine turtle space use within our study site. These findings are generally supported by those of Dwyer *et al*. [44], which found differing space use estimates for dual-tagged crocodiles, but oppose the findings of Babcock *et al*. [45] and Zeh *et al*. [39], which found similar space use estimates for green turtles and dugongs, respectively [39,44,45]. In the present study, satellite telemetry produced larger occurrence and range distribution estimates than acoustic telemetry. As such, treating our results derived from acoustic and satellite telemetry similarly may lead to misinterpretation of turtle movement patterns, and suggests that these methods should not be used interchangeably to address the same ecological questions. Researchers should consider the research objectives, the spatiotemporal structure of the data obtained, biases and limitations (such as acoustic array design), and the associated financial costs of each method prior to selecting a telemetry method or interpreting data from either method.

It is important to compare and consider the temporal scale of data provided by each telemetry method, as increased tracking durations have the potential to reveal additional movement pathways and usage areas, impacting interpretations of space use [71,90,91]. Similar to the findings of Babcock *et al*. [45], both telemetry approaches provided comparable overall tracking durations in this study [45]. Our satellite tracking durations (20 to 234 d) were equivalent to those reported in recent studies tracking juvenile green turtles [92,93]; however, the acoustic tracking durations (8 to 237 d) were considerably shorter than those in the current literature, which have tracked individuals for over a year and even up to 2.5 years [94,95]. Range distributions in particular are heavily influenced by the number of times an individual crosses its range (i.e. effective sample size) [71,96] and space use estimates will generally increase with tracking duration until reaching an asymptote, when the data reflect the full spatial extent used by the individual [97]. Prior marine turtle studies have found home range estimates to stabilize after one to six months of tracking [55,94,98]. The average durations for both acoustic and satellite telemetry in the present study fell within this range and, indeed, there were no meaningful differences in space use for the matching and full temporal duration UDs. This suggests that both telemetry methods provided sufficient tracking durations for space use stabilization of the individuals within this study. While tracking durations were similar for our individuals, transmitter retention should be considered in studies with other species. Satellite transmitters must be attached externally to all taxa for signals to be transmitted, but for most marine species (apart from marine turtles, currently), acoustic transmitters can be surgically implanted, leading to longer retention times [19,99,100].

In addition to the overall temporal duration, it is important to consider the temporal resolution provided by each telemetry method. Because OD estimators interpolate the individual's movements between locations, increased relocation frequency will improve estimates of the accuracy of predicted tracks [71]. Higher relocation frequency can also improve the accuracy of state-space models used to account for location error of satellite telemetry [101]. While the tracking durations were similar between methods, acoustic telemetry provided four times as many raw daily relocations per individual at a much greater frequency. Babcock *et al*. [45] and Dwyer *et al*. [44] reported the same trend from their dual-tagged green turtles and crocodiles, respectively [44,45]. Satellite locations are only transmitted when an individual surfaces, while acoustic signals are transmitted to nearby receivers based on a user-defined interval (in our study, 50–130 s). For species that spend most of their time submerged, such as marine turtles [102,103], this results in a higher frequency of acoustic detections, suggesting that acoustic telemetry may allow for a more accurate depiction of space use and increased certainty of the movement path [45,71].

Acoustic telemetry also provided data at a finer spatial resolution and accuracy than satellite telemetry. Based on receiver detection ranges, the average 50% detection range at our study site was approximately 266 m, and we estimated an average location error radius for COAs to be 216 m. It should be noted, however, that location errors are difficult to assess with certainty due to fluctuating detection probabilities [26,27]. Only 12% of the raw satellite locations collected in this study were classified as LC 0–3. While published errors associated with these classes range from less than 250 m to greater than 1500 m [68], studies have found errors to be greater than these Argos-provided estimates [30,31,104]. This low spatial accuracy associated with Argos satellite telemetry can lead to an overestimation of predicted movement patterns and home range [35]. To compensate for this, we applied an SSM to the satellite tracking data to account for the location errors when estimating occurrence distributions. While similar methods are being developed to incorporate location errors into CTMM models as well [105], the range distributions presented herein do not account for location error so as to remain comparative to the current literature. This inclusion and non-inclusion of location error estimates may in part explain why larger differences were seen between acoustic and satellite RDs than between acoustic and satellite ODs. As the use of Fastloc-GPS transmitters increases, improved satellite tracking data will become more available without the need for extensive post-processing of data [31,35,104]. With average location errors of less than 40 m in stationary tests, it can provide a higher spatial resolution than typical acoustic telemetry [30,33–35].

Interpretations of turtles' individual space use throughout Bimini differed between acoustic and satellite telemetry. Although there was high variability among individuals, with some showing small differences in UD estimates between tracking methods, there was still greater than 81% probability of satellite 95% UDs being larger in size than acoustic UDs. Low BA overlap indices between satellite and acoustic UDs were driven by these size differences, as UDs generally overlapped geographically. The large errors associated with satellite telemetry contributed to the size differences seen in both ODs and RDs [41,91]. The smaller acoustic occurrence distributions observed were also likely due in part to the increased temporal resolution of the acoustic data, suggesting that acoustic telemetry was able to identify movement patterns with fairly high accuracy [71,76]. Smaller acoustic range distributions are likely driven by gaps in acoustic receiver coverage within the array in addition to large satellite telemetry errors.

For all but one turtle, the full temporal duration satellite UDs mostly fell within the MCP of the acoustic array, indicating that the differences in UD estimates between methods were not caused by individuals traveling outside the array and using space beyond its boundaries. Despite not being originally designed to capture the movements of marine turtles, the extent of the acoustic array appears to adequately cover the range of the individuals, indicating that the low density of the array and resulting gaps in coverage contributed to the differences in acoustic and satellite UD estimates [106]. Even in high density arrays, several factors such as benthic substrate, vegetation, salinity, wave action, underwater noise, and more can affect detection probabilities and receiver ranges, sometimes rapidly and unexpectedly [26,27,56]. Any movement or space use within gaps, whether caused by low receiver density or variable detection ranges, will go undetected by acoustic telemetry while still being captured by satellite telemetry, a phenomenon also observed in an exploratory study with a Silvertip shark in the Chagos Islands [19]. Theoretically, since the acoustic array in this study covers most of the ranges of the individuals, acoustic RD estimates should be fairly accurate [106]. However, with such gaps in coverage, it is possible that a portion of the home range is underrepresented. Increasing the density of the array would improve the acoustic space use estimates, potentially leading to smaller differences in the predicted space between telemetry methods. Therefore, a combination of array design and spatiotemporal resolution of the data are likely attributing to the differences seen between acoustic and satellite telemetry. Fluctuating detection ranges as well as the design of an acoustic array introduce inherent bias to space use estimates and careful consideration should be given to array design in relation to the target species and the research question prior to conducting a study [19,25]. Extensive range testing can also improve knowledge of the detection ranges of receivers and allow the incorporation of location errors into analyses [26,27].

While our sample size was small (*n* = 5) and there was high individual variability, our results suggest that the higher temporal and spatial resolution of acoustic telemetry provided a more detailed characterization of the movement paths and predicted space use for the individuals in our study [45]. However, satellite occurrence distribution results should be considered with care, as biases may have been introduced by removing locations over land after the SSM was fit [107]. Although the array design and species’ behaviour of our target population are highly specific, the results from this study suggest that acoustic telemetry may be more appropriate for answering fine-scale ecological questions, such as those related to diel and tidal movement patterns, habitat use within highly heterogeneous benthic substrate, or use of discrete habitats for specific behaviours (e.g. resting spots) [43,61,108–110]. Acoustic telemetry may also be better suited for studies in constrained water bodies (e.g. bays, lakes, rivers) or at study sites with complex coastlines where the larger spatial errors associated with satellite telemetry may produce locations that fall outside the system or over land [107,111]. Additionally, acoustic telemetry can provide insight into intra- or interspecific interactions when individuals are detected simultaneously on the same receiver [37,112,113]. Acoustic transmitters are also available in a range of sizes, some weighing less than 0.5 g, which allows tracking of multiple life stages or species within the same array and can inform multispecies management [50,62,114].

The ability of satellite telemetry to track individuals over an unlimited spatial extent suggests that satellite telemetry is better suited for identifying broad-scale movements and migration pathways [3,93]. Satellite telemetry has captured movements of individuals over thousands of kilometres and spanning ocean basins [36], something that acoustic telemetry cannot capture with the same level of detail, even with the growing number of acoustic arrays and data-sharing networks [38,115]. Satellite telemetry is also advantageous in environments where habitat structures may prevent detections of acoustic signals (e.g. rocky outcrops) or in areas where receiver deployment and data retrieval is difficult and costly (e.g. pelagic ocean and remote areas) [26,27,38].

Our conclusions on the differences in the applicability of each approach are supported by studies that have investigated both fine- and broad-scale information of individual behaviours and population-level space use using both telemetry methods. For example, combining approaches has been used to study habitat associations of marine turtles within foraging areas while also tracking migrations and movements of the same individuals away from these foraging grounds [43,45,116]. The use of acoustic telemetry in addition to satellite tracking has also revealed inter-annual movement and residency patterns of juvenile white sharks and whale sharks [117,118]. Since acoustic and satellite telemetry are each best suited to address distinct ecological questions, combining telemetry approaches within the same study can provide a more complete picture of a species' spatial ecology than either can provide alone. Using both methods can provide knowledge to improve conservation efforts; for example, delineating protected areas that cover habitats used for multiple behavioural phases, such as foraging and migrating [37,116].

When designing a telemetry study, it is important to consider not only the research question at hand, but also the costs associated with each telemetry method in order to efficiently leverage resources [119]. The installation of an acoustic receiver array is a substantial investment of both time and money [38,39]. An acoustic telemetry study requiring array installation is more expensive than satellite telemetry (Argos or Fastloc), regardless of the sample size or study duration tested. Additionally, as array density or extent increases, costs increase as well in regard to not only installation, but maintenance and data retrieval as well. However, provided that they are regularly maintained, the longevity of acoustic arrays can support long-term monitoring efforts and multiple tracking endeavours [109]. Since acoustic arrays can simultaneously track multiple species, this also creates opportunities for resource-sharing among projects using the same receiver array, which may help to offset some of the costs [109]. However, when interpreting data from multi-use arrays, it is important to be aware of biases that may have been introduced if the movement patterns of the target species differ greatly from the species for which the array was originally designed [109].

Barring installation of an array, there is a trade-off between the high costs of individual satellite transmitters and the high costs associated with acoustic array maintenance and data retrieval. Data retrieval is particularly labour-intensive, and costs increase with more remote study sites. At small sample sizes (*n* = 1–5), satellite telemetry is less expensive, with acoustic telemetry becoming the less expensive option as sample size increases beyond that. Because population-level inferences made from an insufficient sample size could misinform conservation measures, while unnecessarily large sample sizes may not be an efficient use of resources, appropriate sample sizes should be determined by the study objectives, movement patterns of the target species, the selected telemetry method, and desired spatial and temporal scales [40,120].

Comparisons of telemetry methods representing different study sites, designs, and species are increasingly necessary as developments in tracking technology offer new opportunities for data collection and lead to increases in the number of studies [14,44]. Collaborative acoustic networks are expanding globally, enabling detections of individuals over increased spatial scales that support tracking along migration pathways [38,115]. Additionally, robotic gliding and animal-borne receivers can now detect individuals in areas where it is difficult to deploy traditional receivers [13,121]. Recent advances in the attachment methods and miniaturization of satellite transmitters have facilitated tracking of individuals previously too small to carry transmitters, providing insight into the cryptic behaviours of neonate turtles [122]. Both acoustic and satellite transmitters can now also be coupled with a suite of sensors to collect *in situ* environmental data, physiological parameters, and more [63,123]. As these developments provide novel ways to elucidate patterns of space use and movement of marine species, comparisons of telemetry methods (such as this study) provide researchers with a growing body of knowledge to inform appropriate tracking methods for specific study goals, as well as to draw appropriate inferences and conclusions from tracking data.

## 5. Conclusion

Given the rapid advances of biotelemetry devices and increasing use of tracking data, it is important to acknowledge that the typical use of acoustic and satellite telemetry may not provide similar estimates of space use for species within foraging areas. This case study provides evidence that each telemetry method provides a different interpretation of space use of the same individuals. We propose that both the spatiotemporal structure of the data, as well as limitations and biases associated with acoustic array design, render each method most ideal for specific purposes. While acoustic telemetry is better suited for assessing fine-scale habitat use, satellite telemetry is better suited for identifying broad-scale movement patterns. This study provides researchers with the information necessary to make an informed decision on which tracking method is best to address the ecological questions at hand. Additionally, this comparison provides the context needed for researchers and managers to properly interpret results from telemetry studies to implement meaningful and successful conservation measures. Using and interpreting telemetry data in the most appropriate way will allow us to improve our knowledge of animal movement patterns and space requirements, resulting in well-informed conservation measures [23].

## Data Availability

Data and associated code are available from Zenodo: https://doi.org/10.5281/zenodo.6863942 [124]. Supplementary material is available online [125].
